# High frequency of *ABCB4* and *ABCB11* gene variants in adult patients with idiopathic chronic or recurrent cholestasis

**DOI:** 10.1097/HC9.0000000000000851

**Published:** 2025-12-16

**Authors:** Paulo Lisboa Bittencourt, Vivian Rotman, Liana Codes, Antônio Ricardo Cárdia Ferraz de Andrade, Raimundo de Araújo Gama, Lívia Geovana Falcão Barbosa Celestino, Raymundo Paraná, Maria Lúcia Gomes Ferraz, Larissa Sampaio de Athayde Costa, Richard J. Thompson, Gilda Porta

**Affiliations:** 1 Escola Bahiana de Medicina e Saúde Pública, Salvador, Bahia, Brazil; 2 Unit of Gastroenterology and Hepatology, Hospital Português da Bahia, Salvador, Bahia, Brazil; 3 Department of Hepatology, Universidade Federal do Rio de Janeiro, Rio de Janeiro, Brazil; 4 Department of Hepatology, Universidade Federal da Bahia, Salvador, Bahia, Brazil; 5 Department of Hepatology, Hospital Aliança—Rede D’Or São Luiz, Salvador, Bahia, Brazil; 6 Department of Hepatology, Universidade Federal de São Paulo, São Paulo, Brazil; 7 Department of Hepatology, Instituto D’Or de Pesquisa e Ensino, Rio de Janeiro, Brazil; 8 Mendelics, São Paulo, Brazil; 9 Institute of Liver Studies, King’s College London, London, UK; 10 Gastroenterology and Pediatrics, Hospital Sírio-Libanês, São Paulo, Brazil

**Keywords:** cholestasis, chronic liver disease, intrahepatic cholestasis of pregnancy, low phospholipid–associated cholelithiasis, progressive familial intrahepatic cholestasis

## Abstract

**Background:**

Several genes encoding proteins essential for normal bile production have been associated with progressive familial intrahepatic cholestasis in children, but there are few studies evaluating the frequency of variants in these genes among adults with chronic cholestatic disease. The aim of this study was to assess frequency of variants in progressive familial intrahepatic cholestasis–associated genes in adults with idiopathic or episodic cholestatic chronic liver disease (cCLD).

**Methods:**

Patients with cCLD of unknown cause followed in 4 different reference centers in Brazil were genotyped for *ABCB11*, *ABCB4*, *ABCC2*, *ATP8B1*, *CFTR*, *JAG1*, *KIF12*, *LSR*, *MYO5B*, *NR1H4*, *PPM1F*, *SERPINA1*, *TJP2*, *USP53*, *VIPAS39*, *VPS33B*, *PEX26*, and *WDR83OS* gene variants using Illumina platforms. Primary biliary cholangitis, primary sclerosing cholangitis, and other causes of cCLD were excluded.

**Results:**

Sixty-five patients (40 females; mean±SD age at disease onset, 26.8±13.4 y) were included. Most (65%) had either a family history of cholestatic liver disease or previous signs and symptoms of intrahepatic cholestasis of pregnancy or low phospholipid–associated cholelithiasis. Fifty-seven (88%) had cCLD, whereas 8 (12%) reported recurrent episodic cholestasis. Some had evidence at diagnosis of cirrhosis (n=20) or hepatocellular carcinoma (n=2) or had undergone liver transplantation (n=10). Sequencing revealed 28 variants in *ABCB4* (n=25) and *ABCB11* (n=3) genes in 42 patients [65%; heterozygous (n=31), homozygous (n=4), and compound heterozygous (n=7)]. Only 17 (61%) were previously reported. Most variants (57%) were classified as pathogenic or likely pathogenic.

**Conclusions:**

More than half of the patients with cCLD of unknown cause exhibited pathogenic or likely pathogenic variants in bile transporter genes, particularly *ABCB4*. Genotyping may identify most of those patients as late-onset patients with multidrug resistance protein 3 deficiency.

## Graphical abstract

**Figure:**
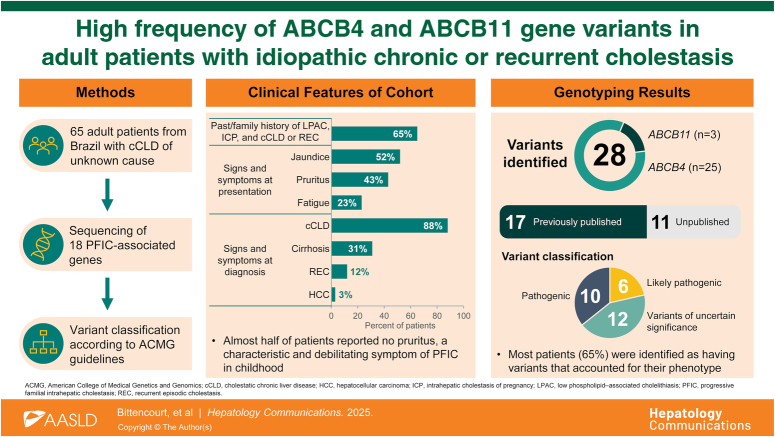

## INTRODUCTION

Genetic cholestasis is a term used to describe a heterogeneous group of liver diseases caused by variants in a multitude of different genes, leading to diseases with different pathobiology, clinical presentation, and prognosis, but with several commonalities due to their cholestatic nature.^1^ Genetic cholestasis was originally described mostly in children and includes inborn errors of metabolism, Alagille syndrome, and progressive familial intrahepatic cholestasis (PFIC).^2^^,^^3^

In PFIC, numerous proteins (and genes) have now been identified, including familial intrahepatic cholestasis protein 1 (FIC1; *ATP8B1*), the bile salt export pump (BSEP; *ABCB11*), and multidrug resistance protein 3 (MDR3; *ABCB4*).^4^^,^^5^ Variants in these 3 genes have also been implicated in the development of recurrent episodic cholestasis (REC) in older children and adults,^2^^–^^4^^,^^6^^,^^7^ often but not always with a benign and fluctuating clinical course.^8^ Likewise, *ABCB4* gene variants were also detected in adult patients with REC, intrahepatic cholestasis of pregnancy (ICP), low phospholipid–associated cholelithiasis (LPAC), and idiopathic cholestatic chronic liver disease (cCLD).^9^ Variants in more recently reported genes, including farnesoid X receptor (FXR; *NR1H4*), tight junction protein 2 (*TJP2*), myosin VB (*MYO5B*), and ubiquitin-specific peptidase 53 (*USP53*), have been associated with at least 13 types of PFIC in children but not in adults.^5^^,^^9^

In adults, *ATP8B1*, *ABCB11*, and *ABCB4* variants have been reported in 18%–34% of patients with REC or idiopathic cCLD.^10^^–^^13^ In a large cohort of German patients with hereditary cholestasis due to FIC1, BSEP, and MDR3 deficiency, 22%, 10%, and 30% of the patients with *ATP8B1*, *ABCB11*, and *ABCB4* variants, respectively, were adults.^14^ Another recent study from the United Kingdom involving 356 patients with adult-onset idiopathic cholestatic liver disease demonstrated that 28% of the patients had either previously described or novel variants in *ABCB4*, *ABCB11*, and *ATP8B1* genes.^15^ Patients with *ABCB4* variants more often had cCLD or ICP, whereas patients with *ABCB11* variants more frequently had ICP or REC.^15^ In contrast to children, most variants observed in adults were in the heterozygous state.^1^^,^^14^^,^^15^

Little is known about the prevalence of variants in *ABCB4*, *ABCB11*, and *ATP8B1* or in other more recently reported PFIC-causing genes, such as *TJP2*, *NR1H4*, *MYO5B*, and *USP53*, in other adult populations. The purpose of the present study was to determine the prevalence of previously described and novel variants of *ABCB4*, *ABCB11*, *ATP8B1*, *TJP2*, *NR1H4*, *MYO5B*, *USP53*, and other genes recently linked to hereditary cholestasis,^16^ including *LSR*, *PPM1F*, *WDR83OS*, *VIPAS39*, and *VPS33B*, in a multiethnic cohort of adult Brazilian patients with either REC or cCLD of unknown etiology.

## METHODS

All patients older than 18 years of age with either REC or cCLD of unknown cause, followed in 4 different reference centers in Brazil, who underwent genotyping for PFIC-causing gene variants, were retrospectively reviewed. Patients were enrolled between October 2022 and December 2023. Participating centers included the Portuguese Hospital of Bahia and the hospitals of the federal universities of Bahia, São Paulo, and Rio de Janeiro.

Cholestasis was defined in the presence of pruritus, with or without jaundice or signs of chronic liver disease (CLD) associated with elevated ALP >1.5 times the upper limit of normal, with or without elevation of GGT and bilirubin.^17^

REC was defined as a past history of 2 or more episodes of cholestasis in a period of more than 6 months, whereas cCLD was defined as signs and/or symptoms of cholestasis lasting more than 6 months without an identifiable etiology.

Evidence of cirrhosis was based on clinical, biochemical, and imaging findings at the local physicians’ discretion, as well as liver histology whenever liver biopsy results were available.

The diagnostic approach to identify the etiology of cryptogenic cCLD in all patients was based on the American Association for the Study of Liver Diseases guidelines.^17^ Briefly, primary biliary cholangitis (PBC) was ruled out using criteria recommended by the American Association for the Study of Liver Diseases. Likewise, primary sclerosing cholangitis (PSC) and small-duct PSC were excluded in all patients through imaging, either MRI or endoscopic cholangiography, or liver biopsy.^18^ Exclusion of other less common causes of cholestasis, such as drug-induced liver disease, alpha-1 antitrypsin deficiency, ductal plate malformations, immunoglobulin G4–related cholangitis, or sarcoidosis, was performed at local investigator discretion based on clinical, laboratory, and histological parameters.^1^^,^^19^

Data obtained from medical records included sex; age at presentation; age at diagnosis; symptoms at presentation, such as pruritus, jaundice, and fatigue; history compatible with LPAC, including cholelithiasis, common bile duct stones, or cholecystectomy with onset before 40 years; prior history of ICP; disease phenotype (REC or cCLD); family history of cholestatic liver disease or consanguinity; signs of cirrhosis at diagnosis; liver enzymes at either presentation or diagnosis, including ALT, AST, ALP, and GGT, and bilirubin; biliary stones or cholecystectomy at imaging; liver histology whenever available; and outcomes such as liver transplantation (LT) and/or death.

Genomic DNA was obtained from a buccal swab in all patients after informed consent. Case selection was limited to 1 individual per family. When more than 1 patient from the same family underwent testing, the first tested family member was selected for inclusion in this study.

All patients underwent sequencing of 18 genes, namely *ABCB11*, *ABCB4*, *ABCC2*, *ATP8B1*, *CFTR*, *JAG1*, *KIF12*, *LSR*, *MYO5B*, *NR1H4*, *PPM1F*, *SERPINA1*, *TJP2*, *USP53*, *VIPAS39*, *VPS33B*, *PEX26*, and *WDR83OS* (Mendelics cholestasis panel). DNA sequencing was performed using Illumina platforms. Base calling was performed using Illumina tools (bcl2fastq). The bioinformatics pipeline followed Broad Institute best practices (https://gatk.broadinstitute.org/hc/en-us/sections/360007226651-Best-Practices-Workflows). After alignment to the reference genome, Genome Reference Consortium Human Reference 38 (GRCh38)/University of California, Santa Cruz human genome 38 (hg38), low-quality and duplicate readings were removed, and variants [single-nucleotide variants (SNVs)/insertion-deletions (indels)] were detected with Genome Analysis Toolkit HaplotypeCaller. Enrichment and analysis concentrated on the coding sequences, flanking intronic regions (±20 bp), and other specific genomic regions previously identified to harbor causative variants. Promoters, untranslated regions, and other noncoding regions were not analyzed. Exonic deletions and copy number variations were identified using ExomeDepth, an R package that estimates the number of copies by comparing the read depth for each target with the mean read depth for the same target using samples from the same sequence library. If a copy number variation was identified, a multiplex ligation-dependent probe amplification assay was employed to confirm the finding.

The variants were listed according to algorithms based on machine learning developed by Mendelics, classified according to the guidelines of the American College of Medical Genetics and Genomics, and described with a nomenclature compatible with the Human Genome Variation Society.^20^ Variants were interpreted as pathogenic (P), likely pathogenic (LP), or variants of uncertain significance (VUS).^20^ All variants were evaluated by a medical geneticist. Frequencies were calculated according to the total number of patients tested.

This study was conducted in accordance with the Declarations of Helsinki and Istanbul. All participants gave written informed consent before enrollment, and the ethics committee of the Federal University of São Paulo approved the study protocol (CAAE 70648123.7.0000.5505).

### Statistical analysis

Dichotomous variables are presented in text and tables as numbers and percentages. Continuous variables are reported as mean±SD or as median (range), respectively, if the distribution was normal or skewed. The software used for analysis was the Statistical Package for the Social Sciences (SPSS Inc., Chicago, Illinois), version 14.0 for Windows.

## RESULTS

Sixty-five patients (40 females; mean±SD age at presentation and diagnosis of 26.8±13.4 y and 38.3±10.2 y, respectively) were included in the study. Clinical and laboratory data of those patients are depicted in Table 1. Ten (25%) of the 40 female patients included in the study had a history of ICP, and 29 (45%) patients had signs and symptoms compatible with LPAC. Ten of 20 (50%) women included in this study who had been pregnant had a history of ICP. Eighteen of 65 (28%) patients also had a family history of liver disease. Approximately half experienced pruritus (43%) or jaundice (52%) at disease onset. Most (88%) of the patients had cCLD at diagnosis. Twenty (31%) and 2 (3%) patients had developed cirrhosis and hepatocellular carcinoma (HCC), respectively, and 10 (15%) required LT due to complications of cirrhosis (n=9) or HCC (n=1). Cirrhosis was diagnosed based on clinical, laboratory, and imaging features (n=12) or histology (n=8).

**TABLE 1:** Clinical and laboratory features of patients (N=65)

Clinical and laboratory data	
Sex, female, n (%)	40 (62)
Age at presentation, mean±SD	26.8±13.4
Age at diagnosis, mean±SD	38.3±10.2
History of ICP in female patients, n (%)	10 (25)
History compatible with LPAC syndrome, n (%)	29 (45)
Family history of liver disease, n (%)	18 (28)
Past or family history compatible with LPAC, ICP, and cCLD or REC, n (%)	42 (65)
Signs and symptoms at presentation	
Pruritus, n (%)	28 (43)
Jaundice, n (%)	34 (52)
Fatigue, n (%)	15 (23)
Signs and symptoms at diagnosis	
REC, n (%)	8 (12)
cCLD, n (%)	57 (88)
Cirrhosis, n (%)	20 (31)
Hepatocellular carcinoma, n (%)	2 (3)
Liver transplantation, n (%)	10 (15)
Laboratory findings at diagnosis	
AST (×ULN) median (range)	3.6 (0.8–17.4)
ALT (×ULN) median (range)	4.2 (0.6–25.1)
ALP (×ULN) median (range)	2.2 (0.5–11.5)
GGT (×ULN) median (range)	4.2 (0.2–48.1)
Bilirubin (×ULN) median (range)	1.1 (0.3–31.0)

*Note:* Data are presented as mean±SD or number (percentage) or median (range) whenever the distribution is skewed.

Abbreviations: cCLD, cholestatic chronic liver disease; ICP, intrahepatic cholestasis of pregnancy; LPAC, low phospholipid–associated cholelithiasis; REC, recurrent episodic cholestasis; ×ULN, number of times the upper limit of normal.

Most of the patients had 2–3-fold elevations of AST, ALT, and ALP at diagnosis, and more marked abnormalities in GGT were encountered in those patients (Table 1).

Genotyping revealed 28 variants in *ABCB4* (n=25) and *ABCB11* (n=3) genes in 42 (65%) patients. Only 17 (61%) of those 28 variants have been previously reported. Most (57%) variants were P or LP, and more than half of them were in the heterozygous state (Tables 2 and 3). No other variants were encountered in other genes. Briefly, 38 of those 42 patients had cCLD, while 4 had REC, 16 had evidence of cirrhosis, 2 had HCC, and 8 underwent LT. Genotype–phenotype correlations are depicted in Table 3.

**TABLE 2:** Genotyping of patients with unexplained cholestasis

	n (%)
Identified variants	
Number of different variants	28 (100)
Number of previously published variants	17 (61)
Predicted pathogenicity of variants	
VUS	12 (43)
LP	6 (21)
P	10 (36)
Number of variants in affected patients	
1	31 (72)
2	11 (26)
3	1 (2)
Type of variant	
Frameshift	3 (11)
Missense	18 (64)
Intronic	3 (11)
Nonsense	3 (11)
Synonymous	1 (4)

Abbreviations: LP, likely pathogenic; P, pathogenic; VUS, variant of uncertain significance.

**TABLE 3:** Clinical data according to *ABCB4* and *ABCB11* variants

Genotype	Age at onset	Sex	Family history	Cirrhosis and/or LT	Phenotype
*ABCB4*					
p.(Ala1220Asp)/p.(Ile1157Met)	22	Male	+	+	cCLD
p.(Ala934Thr) +/+	34	Female	−	+	cCLD, LPAC
p.(Ala934Thr) +/+	25	Female	−	+	cCLD, LPAC
p.(Ala934Thr) +/−	26	Male	−	−	cCLD, LPAC
p.(Ala934Thr) +/−	39	Female	+	−	cCLD, LPAC
p.(Arg1046Ter) +/−	21	Female	−	−	cCLD, LPAC
p.(Arg47Gln)/p.(Ala934Thr)	24	Female	+	−	ICP, cCLD
p.(Arg582Trp) +/−	61	Male	−	−	cCLD, LPAC
p.(Arg582Trp) +/−	27	Female	−	−	cCLD
p.(Arg582Trp) +/−	41	Female	+	−	cCLD
p.(Arg582Trp) +/−	21	Female	+	+	ICP, cCLD, LPAC
p.(Arg582Trp)/p.(Gln772Lys)	29	Female	+	+	cCLD
p.(Arg590Gln) +/−	37	Female	−	−	cCLD
p.(Arg590Ter) +/−	13	Female	−	−	cCLD
p.(Gln572Ter) +/−	59	Male	−	−	cCLD, LPAC
p.(Gln945HisfsTer21) +/−	53	Female	−	−	cCLD
p.(Gly432Asp) +/−	23	Female	−	−	cCLD
p.(Gly535Asp)/Ile1p.(Arg590Gln)	50	Female	+	−	cCLD
p.Gly954Ser +/−	17	Female	+	+	cCLD
p.(Ile1242AsnfsTer47) +/−	41	Female	+	+	ICP, cCLD, LPAC
p.(Ile1242AsnfsTer47) +/−	18	Female	+	−	ICP, cCLD, LPAC
p.(Ile1242AsnfsTer47)/p.Arg590Gln	23	Male	−	+	cCLD, LPAC, HCC
p.(Ile1242AsnfsTer47)/p.(Ala934Thr)	33	Male	+	+	cCLD
p.(Ile1242AsnfsTer47)/p.(Ala934Thr)	20	Male	+	+	cCLD
p.(Ile1242AsnfsTer47)/p.(Arg494His)	25	Female	−	+	cCLD, LPAC
p.(Ile460Thr) +/−	31	Female	−	+	cCLD, HCC
p.(Lys236=) +/−	29	Female	−	−	REC
p.(Pro1044Leu)/p.(Ser755Tyr)	27	Female	−	+	cCLD, LPAC
p.(Pro1044Leu)/p.(Ala934Thr)/p.(Ser755Tyr)	22	Male	−	−	cCLD, LPAC
p.(Ser320Phe) +/+	22	Male	−	+	cCLD, LPAC
p.(Ser339PhefsTer17) +/−	23	Female	+	−	ICP, cCLD
p.(Ser339PhefsTer17) +/−	47	Female	+	−	cCLD
p.(Ser339PhefsTer17) +/−	51	Male	+	−	cCLD
p.(Ser339PhefsTer17) +/−	28	Male	−	−	cCLD
p.(Thr715lle) +/−	36	Female	+	−	ICP, REC
c.2682+1G>A +/−	18	Female	−	−	cCLD, LPAC
c.2682+1G>A +/−	23	Female	+	+	ICP, cCLD, LPAC
c.2784-12T>C +/−	18	Male	−	−	cCLD, LPAC
c.2784-12T>C +/−	31	Female	−	−	REC
c.1006-1G>T/p.Arg590Gln	55	Female	+	−	ICP, cCLD, LPAC
*ABCB11*					
p.(Arg1050Cys) +/+	16	Male	−	−	REC
p.(Arg1231Gln)/p.(Val1112Phe)	19	Male	−	−	cCLD

Abbreviations: cCLD, cholestatic chronic liver disease; HCC, hepatocellular carcinoma; ICP, intrahepatic cholestasis of pregnancy; LPAC, low phospholipid–associated cholestasis; LT, liver transplantation; REC, recurrent episodic cholestasis.

The most common *ABCB4* variants observed were p.(Ala934Thr), p.(Ile1242AsnfsTer47), and p.(Arg582Trp) (Table 4). Three different variants were found in the *ABCB11* gene in 2 patients: 1 with REC was homozygous for p.(Arg1050Cys), and the other with cryptogenic CLD had 2 variants, p.(Val1112Phe) and p.(Arg1231Gln).

**TABLE 4:** Variants identified in patients with unexplained cholestasis (N=65)

Variant	Type	n	Previously reported variant	Interpretation of pathogenicity	Allelic frequency (%)
*ABCB4*					
p.(Ala1220Asp)	Missense	1	−	VUS	1.54
p.(Ala934Thr)	Missense	10	+	VUS	15.38
p.(Arg1046Ter)	Nonsense	1	+	P	1.54
p.(Arg47Gln)	Missense	1	+	LP	1.54
p.(Arg494His)	Missense	1	+	VUS	1.54
p.(Arg582Trp)	Missense	5	+	P	7.69
p.(Arg590Gln)	Missense	4	+	VUS	6.15
p.(Arg590Ter)	Nonsense	1	+	P	1.54
p.(Gln572Ter)	Nonsense	1	−	P	1.54
p.(Gln772Lys)	Missense	1	−	VUS	1.54
p.(Gln945HisfsTer21)	Frameshift	1	−	LP	1.54
p.(Gly432Asp)	Missense	1	−	VUS	1.54
p.(Gly535Asp)	Missense	1	+	LP	1.54
p.(Ile1157Met)	Missense	1	−	VUS	1.54
p.(Ile1242AsnfsTer47)	Frameshift	7	−	P	10.77
p.(Ile460Thr)	Missense	1	−	VUS	1.54
p.(Lys236=)	Synonymous	1	−	VUS	1.54
p.(Pro1044Leu)	Missense	2	+	VUS	3.08
p.(Ser320Phe)	Missense	2	+	P	3.08
p.(Ser339PhefsTer17)	Frameshift	3	+	LP	4.62
p.(Ser755Tyr)	Missense	2	+	VUS	3.08
p.(Thr715lle)	Missense	1	+	LP	1.54
c.2682+1G>A	Intronic	2	+	P	3.08
c.2784-12T>C	Intronic	2	+	LP	3.08
c.1006-1G>T	Intronic	1	−	P	1.54
*ABCB11*					
p.(Arg1050Cys)	Missense	2	+	P	3.08
p.(Val1112Phe)	Missense	1	−	VUS	1.54
p.(Arg1231Gln)	Missense	1	+	P	1.54

Abbreviations: LP, likely pathogenic; P, pathogenic; VUS, variant of uncertain significance.

Comparison of clinical and laboratory data of patients who tested positive with their counterparts who tested negative for disease-causing variants revealed that the former patients tended to be younger, have less fatigue, and have higher AST and ALT levels at diagnosis (Supplemental Table S1, http://links.lww.com/HC9/C185).

## DISCUSSION

Few studies have evaluated the frequency of cholestasis-related gene variants in adult patients with cryptogenic CLD or REC.^10^^–^^14^ All of them were conducted in European patients, and little is known about the frequency of variants in other adult populations. In this study, *ABCB4* and *ABCB11* gene variants were encountered in more than two-thirds of the Brazilian patients with idiopathic cCLD or REC. Most of them were classified as P or LP. This frequency is much higher compared with previous reports.^10^^–^^15^ In this regard, *ABCB4* and *ABCB11* variants have been reported in 18%–34% of adult patients with idiopathic cCLD in different small cohorts from France,^10^ Italy,^11^^,^^12^ and Denmark.^13^ In a larger cohort of 427 patients with suspected inherited cholestasis from Germany, 149 (35%) patients were shown to have *ATP8B1*, *ABCB11*, and *ABCB4* gene variants. Detailed clinical data from those patients were not reported by the authors.^14^ In addition, Nayagam et al.^15^ reported data from 356 patients with ICP, REC, or CLD who underwent *ABCB4, ABCB11*, or *ATP8B1* genotyping in the United Kingdom. The authors found variants in *ABCB4* (n=46), *ABCB11* (n=35), and *ATP8B1* (n=28) in 28% of those patients. Like our findings, most *ABCB4* variants were in the heterozygous state and were classified as P or LP. In this study,^15^ *ABCB4* variants were more frequently associated with cCLD or ICP, whereas *ABCB11* and *ATP8B1* gene variants were more frequently found, respectively, in patients with ICP and REC and patients with other clinical diagnoses for CLD.

In the current study, VUSs in the *ABCB4* gene were found in 19 of 42 (45%) patients in whom variants were identified. In this respect, the most common variant encountered in our patients with cCLD with or without a past history of LPAC was p.(Ala934Thr). This is still considered a VUS due to its relatively high allelic frequency in populations of African ancestry, but recent data from Spain, the United Kingdom, and France link the p.(Ala934Thr) variant to chronic cholestasis and/or LPAC.^15^^,^^21^^–^^23^ When comparing Native American and European ancestries, it is important to highlight that variant classification is a dynamic process, and variant classifications are subject to change based on the evolving body of scientific knowledge. This underscores the necessity of regular reclassification of variants, including those designated as VUSs initially, to ensure clinical decisions are grounded in the most current evidence available. Reduced protein levels in functional studies^21^ with p.(Ala934Thr) indicate that the variant may be reclassified as LP or P. Of note, this variant was possibly seen at a higher frequency in our cohort of patients due to the ethnic background of the Brazilian population, with a higher percentage of African and Native American ancestries compared with Europeans.

Most of the patients in the present cohort had cryptogenic cCLD. Almost half of them had signs or symptoms of cirrhosis or a requirement for LT at diagnosis. Hereditary cholestasis was suspected in most of them due to disease onset in early adulthood and biochemical and/or histological signs of cholestasis, since approximately half of them never reported pruritus.

Prolonged delay in diagnosis may be attributed to lower awareness of hereditary cholestasis as a cause of adult-onset cholestatic liver disease and lack of access to genetic testing in Brazil. When compared with their counterparts without disease-causing variants, patients with either *ABCB4* or *ABCB11* variants tended to be younger, have less fatigue, and have higher levels of AST and ALT at diagnosis. However, these results should be interpreted with caution and may require confirmation through studies with a larger and more representative sample size.

This study has limitations, including its sample size, lack of bile acid measurements, and the employment of targeted sequencing instead of whole exome or whole genome sequencing; however, our data reinforce the suggestion that genetic testing, as recently recommended in the European Association for the Study of the Liver guidelines,^1^ should be offered to adults with unexplained cCLD or REC, particularly after exclusion of PBC and PSC or whenever genetic cholestasis is suspected.

In conclusion, more than half of the Brazilian patients with cCLD or REC have variants in the *ABCB4* and *ABCB11* genes. Genetic testing provided a more accurate diagnosis of hereditary cholestasis in those patients.

## Supplementary Material
