# Supplementary material for: High frequency of ABCB4 and ABCB11 gene variants in adult patients with idiopathic chronic or recurrent cholestasis
Source: Hepatol Commun. 2025 Dec 16;10(1):e0851. doi: 10.1097/HC9.0000000000000851 (PMC12708102; doi:10.1097/HC9.0000000000000851)
Supplement: Supplementary file 1 [file hc9-10-e0851-s001.pdf]

High Frequency of ABCB4 and ABCB11 Gene Variants in Adult Patients With Idiopathic Chronic or Recurrent Cholestasis

Paulo Lisboa Bittencourt, MD,[1,2] Vivian Rotman, MD, PhD,[3] Liana Codes,[1,2] Antônio Ricardo Cárdia Ferraz de Andrade, MD, PhD,[2,4,5] Raimundo de Araújo Gama,[6] Lívia Geovana Falcão Barbosa Celestino, MD,[6] Raymundo Paraná, MD,[5,7] Maria Lúcia Gomes Ferraz, MD,[6] Larissa Sampaio de Athayde Costa, MD,[8] Richard J. Thompson, MD,[9] Gilda Porta, MD[10]

[1]Escola Bahiana de Medicina e Saúde Pública, Salvador, Bahia, Brazil; [2]Unit of Gastroenterology and Hepatology, Hospital Português da Bahia, Salvador, Bahia, Brazil; [3]Universidade Federal do Rio de Janeiro, Rio de Janeiro, Rio de Janeiro, Brazil; [4]Universidade Federal da Bahia, Salvador, Bahia, Brazil; [5]Hospital Aliança - Rede D'Or São Luiz, Salvador, Bahia, Brazil; [6]Universidade Federal de São Paulo, São Paulo, São Paulo, Brazil; [7]Instituto D'Or de Pesquisa e Ensino, Rio de Janeiro, Rio de Janeiro, Brazil; [8]Mendelics, São Paulo, São Paulo, Brazil; [9]Institute of Liver Studies, King's College London, London, United Kingdom; [10]Hospital Sírio-Libanês, São Paulo, São Paulo, Brazil

Supplemental Table S1. Clinical and Laboratory Features of Patients Based on Presence of Disease-Causing Variants (N=65)

**Clinical and laboratory data**	**Tested positive (n=42)**	**Tested negative (n=23)**	***p***
Sex, female, n (%)	28 (67)	12 (52)	0.25
Age at diagnosis, mean ± SD	30.4 ± 12.8	36.7±14.5	0.076
History of ICP in female patients, n (%)	8 (29)	2 (9)	0.31
History compatible with LPAC syndrome, n (%)	20 (48)	9 (39)	0.51
Family history of liver disease, n (%)	13 (31)	5 (22)	0.57
Past or family history compatible with LPAC, ICP, and cCLD or REC, n (%)	30 (71)	12 (52)	0.66
**Signs and symptoms at presentation**			
Pruritus, n (%)	21 (50)	7 (30)	0.10
Jaundice, n (%)	23 (55)	11 (48)	0.61
Fatigue, n (%)	6 (14)	9 (39)	0.035
**Signs and symptoms at diagnosis**			
REC, n (%)	5 (12)	3 (13)	1.00
cCLD, n (%)	37 (88)	20 (87)	1.00
Cirrhosis, n (%)	15 (36)	5 (22)	0.24
Hepatocellular carcinoma, n (%)	2 (5)	0 (0)	0.54
Liver transplantation, n (%)	8 (19)	2 (9)	0.31
**Laboratory findings at diagnosis**			
AST (x ULN) median (range)	4.1 (1.0-17.4)	2.7 (0.8-7.8)	0.01
ALT (x ULN) median (range)	5.4 (0.6-25.1)	2.5 (0.8-7.8)	0.006
ALP (x ULN) median (range)	2.1 (0.5-11.5)	2.8 (0.7-6.2)	0.91
GGT (x ULN) median (range)	4.3 (0.2-48.1)	3.5 (0.8-19.8)	0.27
Bilirubin (x ULN) median (range)	1.2 (0.3-31.0)	1.0 (0.3-18.0)	0.57

cCLD, cholestatic chronic liver disease; ICP, intrahepatic cholestasis of pregnancy; LPAC, low phospholipid–associated cholelithiasis; REC, recurrent episodic cholestasis; x ULN, number of times the upper limit of normal.

Data are presented as mean ± SD or number (percentage) or median (range) whenever the distribution is skewed.
